# Viral Instant Mutation Viewer: A Tool to Speed Up the Identification and Analysis of New SARS-CoV-2 Emerging Variants and Beyond

**DOI:** 10.3390/v15081628

**Published:** 2023-07-26

**Authors:** Vincent Wilde, Bruno Canard, François Ferron

**Affiliations:** 1 Architecture et Fonction des Macromolécules Biologiques, CNRS-UMR 7257, Polytech Case 925, 13009 Marseille, France; vincent.wilde@univ-amu.fr (V.W.); bruno.canard@univ-amu.fr (B.C.); 2 Marseille and Laboratoire Architecture et Fonction des Macromolécules Biologiques (AFMB), Aix-Marseille Université, AFMB UMR 7257, 13288 Marseille, France; 3 European Virus Bioinformatics Center, Leutragraben 1, 07743 Jena, Germany

**Keywords:** genomics, proteomics, Django, mutation, variants, virus bioinformatics, software, SARS-CoV-2

## Abstract

The appearance of genetic variants impacts vaccination efficiency and therapeutic options, generating a need to map and relate mutations observed in the proteome and the genome. We develop an user-friendly web service software (Viral Instant Mutation Viewer or VIMVer) which allows a direct identification of mutations in the genome and its counterpart in the viral proteome. Since its emergence in 2019, the severe acute respiratory syndrome coronavirus-2 (SARS-CoV-2), responsible for the COVID-19 pandemic, has generated an overwhelming amount of data while becoming one of the most studied viruses of the Nidovirales order. We originally developed this tool during the COVID pandemic; thus, for any SARS-CoV-2 nucleotide sequence, the web service gives a fast identification, mapping, and display of new mutations simultaneously at the nucleotide and amino acid level in comparison to a reference sequence (Wuhan-1). Furthermore, the lineage or the relative position to the known lineage of the variant of interest is available on the link to Phylogenetic Assignment of Named Global Outbreak LINeages (PANGOLIN COVID-19). The workflow presented here is available online. The source code is released under public license and can be easily adapted for further development to other viruses.

## 1. Introduction

Coronaviruses (CoVs) are large-genome, positive-strand RNA viruses of the Nidovirales order that have recently attracted global attention due to the ongoing COVID-19 pandemic. Despite significant efforts to control its spread through worldwide vaccination campaign strategies, SARS-CoV-2 is still causing substantial health and economic burden, emphasizing the need for a continuous monitoring of its genome evolution to identify the circulating and emerging SARS-CoV-2 lineages [1]. Thus, the scale of the COVID-19 pandemic has led to unprecedented efforts by the research community to rapidly identify variant sequences, to test therapeutics and vaccines, and to understand the molecular basis of SARS-CoV-2 entry, pathogenesis, and immune targeting in light of these emerging mutations. This effort resulted in the availability of more than 15 million genomic sequences available through the GISAID [2] data portal: “https://www.gisaid.org/ (accessed on 30 June 2023)”. Whole-genome sequencing allows us to discriminate one lineage from the other and spot variants of concern (VOCs). Next-generation sequencing (NGS) methods are widely used, from hospitals to academic research labs, and yet the process to rapidly identify mutant sequences and its impact at the protein level is still tedious, although maybe less so since the development of tools like nextclade [3]. In this work, we present the Viral Instant Mutation Viewer (VIMVer) workflow, which allows the user to easily identify the mutation hot spots on the genome, to assign its lineage, and to instantly visualize its potential impact at the protein level. This resource can help practitioners and researchers quickly discriminate the new emergence of mutations at the genome level and its potential impact at the protein level. VIMVer is publicly available at: “https://vimver.afmb.univ-mrs.fr/” (accessed on 21 September 2022).

## 2. Materials and Methods

Our goal is to propose a tool to quickly analyze viral genomes and to rapidly detect mutations and their consequences on the protein structure relative to a reference strain present in the database. The VIMVer workflow is developed under Ubuntu 22.04 LTS and is an early-stage web service developed in Python 3.8+ using the Django framework. It is intended to be friendly to the virology community. The Django development framework has a heavy and rigorous but adaptable architecture which simplifies any integration of new functions during development.

**Figure 1:**
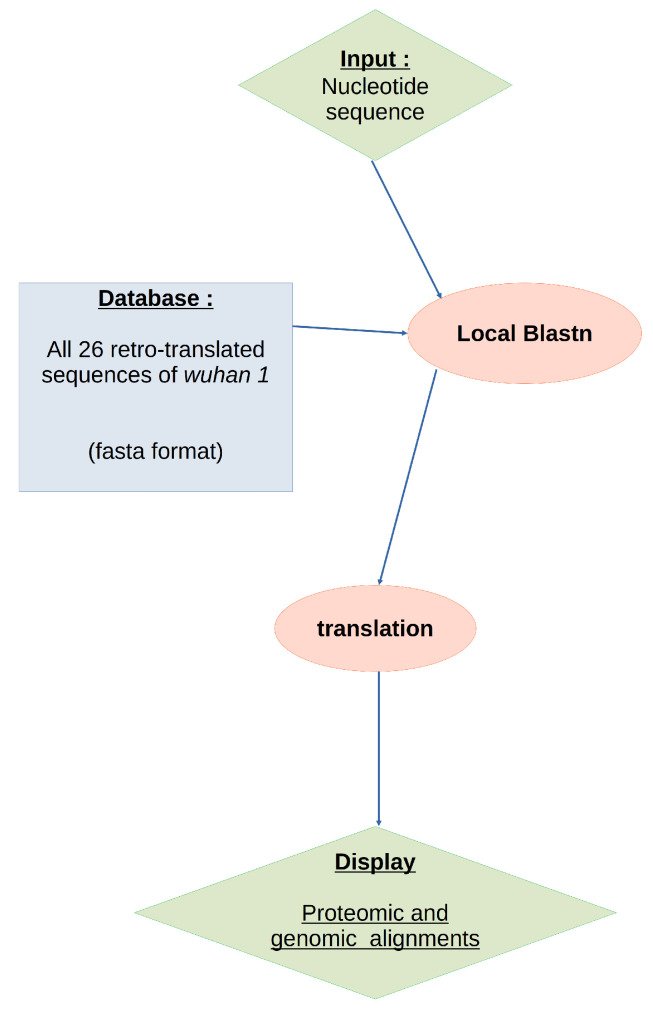
The workflow scheme of VIMVer.

The workflow is presented in Figure 1 and can be summarized as follows. A nucleic sequence is submitted as an input to a search engine, currently a blastn [4], to query the VIMVer reference database. The multiple sub-alignments generated by blastn are optimized through a realign procedure using Muscle version 3.8 [5] to proof the alignment. All alignments are carried out using the nucleotide sequences and are dynamically translated to generate the corresponding protein alignment. The protein alignment reflects the genomic variability and is independent of amino acid composition. The final step is the dynamic visualization of both alignments (nucleotide/amino acids), which allows us to spot mutations, whether silent or not. The user can submit the resulting output to the PANGOLIN webserver to assess lineage.

### 2.1. Database

Any viral reference sequence could be implemented in the database and constituted of the retro-translated genomic sequence(s) from the corresponding viral protein sequences. In this context, viral genome sequences are treated as modular sequences representing their translated states. Therefore, genomes coding for polyproteins, which are subsequently processed into multiple proteins, are pre-annotated at the genomic level. Currently, the development focus of VIMVer lies in the SARS-CoV-2 genome. Consequently, the database currently consists of the complete SARS-CoV-2 genome (Wuhan-1: NC_045512.2 or YP_009725297.1 from NCBI), encompassing 26 proteins in both nucleotide and amino acid sequences. Each sequence type is organized into separate files in fasta format. To accomplish this, the entire genome was divided into sub-sequences to match each functional protein. These nucleotide sub-sequences were meticulously retro-translated to ensure the preservation of codon-wise reading frames and to address ribosomal frameshifts, a characteristic feature of CoV genomes. Notably, the first open reading frame (ORF) of CoV involves a ribosomal frameshift, leading to the synthesis of two polyproteins, pp1a and pp1ab [6]. This frameshift has implications for the translation of the landmark protein nsp12, as its N-terminal is encoded at the end of ORF1a, overlapping nsp11, while the remaining coding sequence is shifted by +1 frameshift. In our local database, we have merged nsp11 at the beginning of nsp12 to align with the coding reading frame, resulting in the duplication of the corresponding retro-translated sequence’s nth nucleotide. Therefore, both genomic and protein nsp12 sequences are accurately assigned and numbered in our database.

Furthermore, Django utilizes the MySQL database to store all items from the created models, including user request results, which are only accessible for 24 h. VIMVer generates fasta files containing the resulting alignments.

### 2.2. Search Engine and Alignments

As shown in Figure 1, we are using blastn [4] mainly to confront the query to our database. Blastn parameters are left by default. In the event that the inquiry is too distant from the reference or the inquiry’s sequence has too many ‘N’s (unspecified nucleotide(s)), blastn produces multiple sub-alignments for a hit. Muscle [5] (v3.8) is used to correct the alignment [7]. All alignments are carried out with respect to the reference nucleotide sequences to serve as anchors for the following codon-wise co-translation. Here blastn was selected for its ability to deploy and parameterize, considering the size of the database.

The output is a list of as many hits as blastn found for the maximum sequences of the prepared dataset. Each hit opens a two-frame window that shows on the left panel the protein alignments and on the right panel the nucleotide alignments. Sequences are interactive, so one can follow its position on the sequence using the mouse cursor on the corresponding sequence. Mutations are highlighted by an asterisk under each alignment. Outputs are downloadable in multi-fasta format.

### 2.3. Accessibility

Each job runs under few seconds and is identified by a key made of 6 printable characters, generated when a query is launched. This job name allows the users to retrieve their run within 24 h. Additional information is available on the project’s GitHub.

### 2.4. Deployment

Viral Instant Mutation Viewer is deployed on a apache2 server hosted from our laboratory server. The ssl protocol was obtained with Encrypt’s services. For users or developers who want to install VIMVer locally, the source code is freely available; however, it will not be functional straight away after download. Indeed the settings and configurations file has to be protected. A setting file (setting.py) including an encryption key used to run Django functionality needs to be present in the path. This setting file is generated once by the administrator when creating the VIMVer project within Django. Moreover, it is also within the setting file that the administrator will define its server setting prior to deployment. Details for installation are provided in the GitHub repository.

## 3. Results

### 3.1. Web Interface and Practical Example

VIMVer is a bioinformatics tool that quickly shows mutations on the user’s query with the proper numbering both in genome and protein. Our goal is to propose a tool to quickly analyze viral genomes, and for any SARS-CoV-2 sequence of interest, VIMVer rapidly detects mutations and their consequences on the protein structure relative to the Wuhan-1 reference strain. This is particularly important in the case of the frameshift, which obscures the recognition of the 1b fragment. Our web tool is composed of three pages named query, finder, and viewer. VIMVer is accessible through the internet using any web browser.

**Figure 2:**
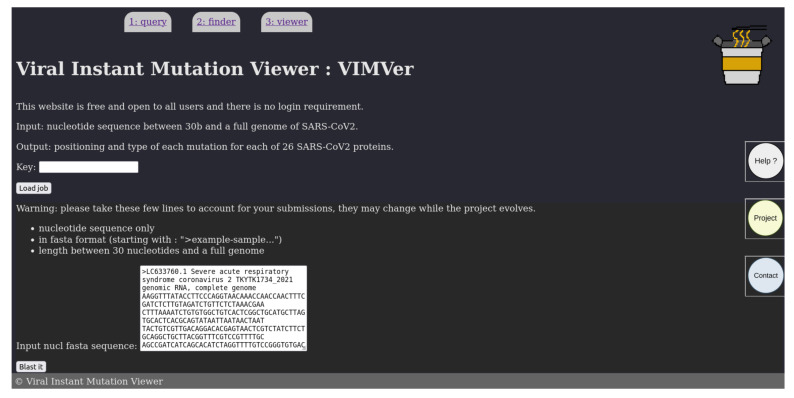
Snapshot of the input form page of VIMVer. A fasta sequence with the header can be pasted into the form section as query.

The user can paste his query in fasta format on the homepage, shown in Figure 2. The inquiry must be a nucleotide sequence possibly from a sequencing result in fasta format, while the length can range from a random recommended minimum of 30 nucleotides, or a single coding sequence, to a whole viral genome. In this version, queries have to be SARS-CoV- or SARS-CoV-2-related to retrieve hits from blastn. When the query is submitted, VIMVer generates a job-key, which is only shown once. On the same page, there is a link to the PANGOLIN [8] web service available though: “https://pangolin.cog-uk.io/” (accessed on 3 May 2023), to retrieve information to determine the lineage of the query sequence.

**Figure 3:**
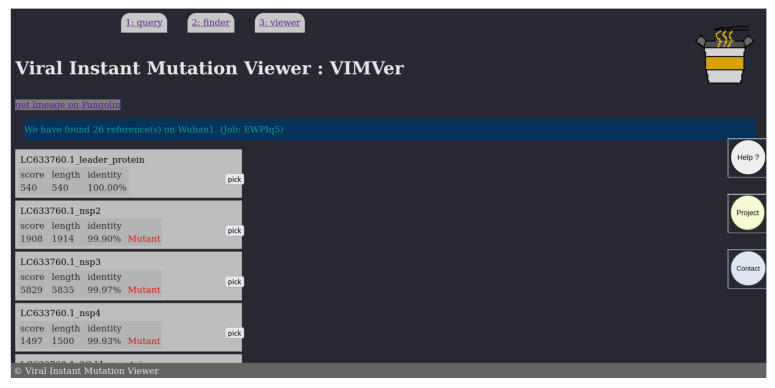
Snapshot of the resulting VIMVer search. The hits are presented as a list with an identification number corresponding to the identified domain. The score, the matching length, and the sequence identity to the reference sequence are shown. If non-silent mutations are detected, then a Mutant mention appears in red. A pick button allows us to access individual results. Response time around 1 s.

**Figure 4:**
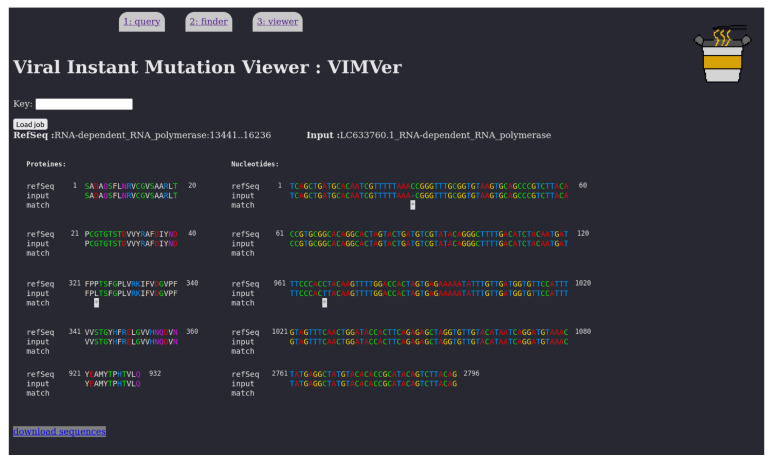
VIMVer result page presenting a dual alignment of the GISAID entry EPI_ISL_14769247 on nsp12 Wuhan reference protein (**left**) and genome (**right**). The alignment is partial due to space reasons and was shortened from positions 81 to 281. Shown are protein numbering 1 to 40, 321 to 360, and 921 to 932. Shown are nucleotide numbering 1 to 120, 961 to 1080, and 2761 to 2796. On the left we have the protein side (20 residues per line), on the right the nucleotide side (60 residues per line). Any mutations are marked by ‘*’. The mouse cursor is dynamic on both alignments, so the user can highlight the amino acid/codon pair. Response time around 2 s.

The result, shown in Figure 3, is returned as a table for which each entry corresponds to a “blastn” hit, described by the score, length, and identity percentage. Considering that our database is small, the E-value is not relevant and therefore not shown, albeit calculated; instead, the score is shown, as it represents the number of aligned residues, which allows us to infer the identity percentage. If the query nucleotide sequence is not a silent mutation, then the corresponding hit is marked as “Mutant” in red. At the end of the page, the user can download the blastn results in fasta format. To access the resulting alignment sampled in Figure 4, click on the ‘pick’ button on the same line.

The VIMVer result page presents a dual alignment of the query against the reference sequence. The left alignment corresponds to the aligned proteins, while the genome alignment is on the right. Any mutations are marked by an asterisk. And all the codons or the amino acids of the same position are correlated when one is hovered over with the mouse cursor. Nucleic residues are colored by the following rule: A red, C green, G yellow, T blue, while proteic residues are colored by hydrophobicity logic as follows: hydrophobic (AILPVM) white, small polar (CSTG) green, positively charged (RHK) blue, negatively charged (DE) red, aromatic (FWY) pink, amphiphilic (NQ) purple.

### 3.2. Benchmark

As proof of concept, we tested our project in a real-life scenario. To do so, we looked for two well-known proteins: the domain RdRp and the spike protein S.

Indeed, the S protein is the third ORF of the SARS-CoV-2 genome, and the gene most prone to accumulating mutations that influence the infectivity of emerging variant [9]. If one wants to observe and analyze the spike nucleotide and protein sequence evolution straight out of the sequencing campaign or from database analysis, VIMVer makes the task easy and quick.

The nsp12 gene (the main replicative RdRp core) is also a point of interest for our project since it is often reported with incorrect numbering due to the Nidovirales frameshift [6,10].

As an example, a random entry in GISAID (EPI_ISL ID: 14769247) is selected and examined for mutations. We selectively focus on the RdRp domain for the frameshift management and the spike protein for its high mutation susceptibility. The goal is to process a whole genome, which is why the test sequence has to be a complete genome with a high coverage rate. On the query page of VIMVer, a single fasta sequence was pasted in the input text box.

**Figure 5:**
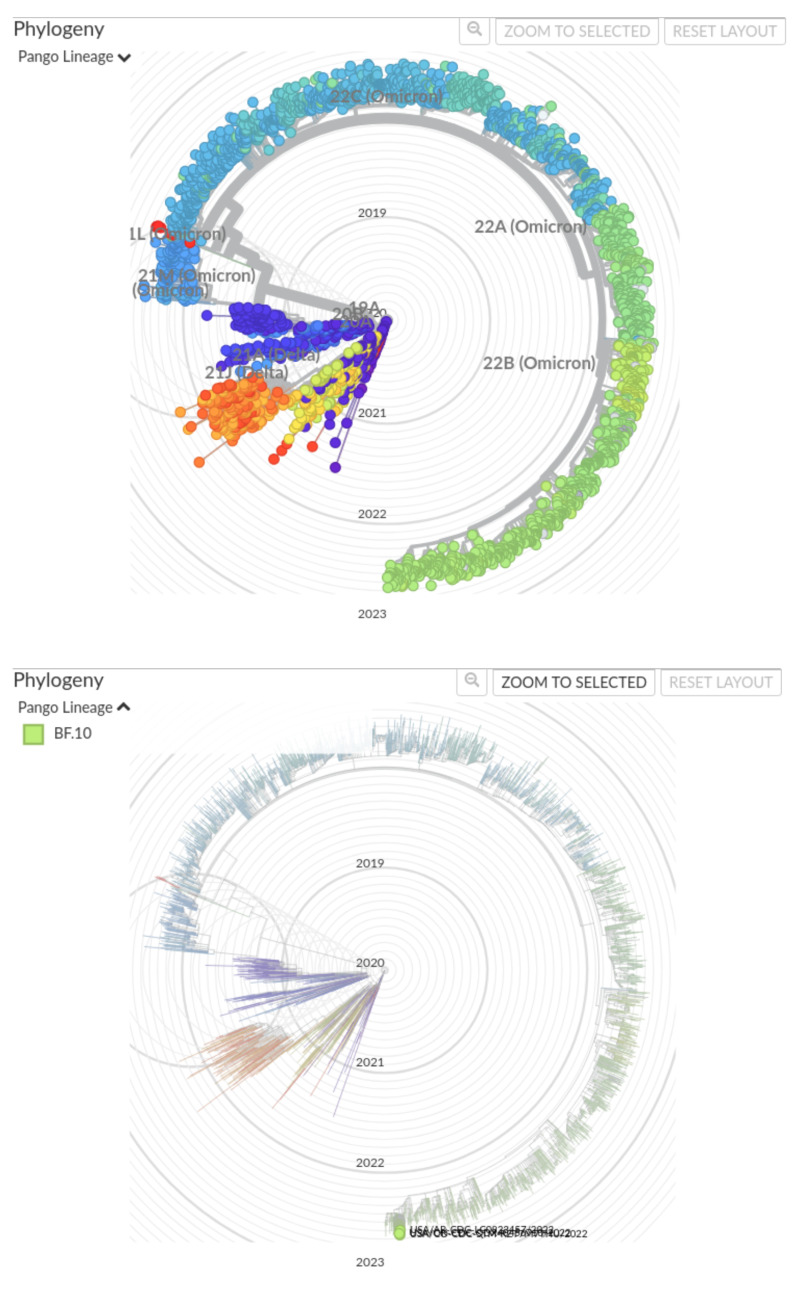
Position of lineage BF10 on the most recent SARS-COV-2 phylogeny (at the date of the writing of the paper): “Genomic epidemiology of SARS-CoV-2 with subsampling focused globally over the past 6 months” (Dataset: ncov, open, global, 6 m; Data range: from 17 December 2019 to 4 September 2022; data form GenBank), carried out on nextstrain.org at “https://nextstrain.org/ncov/open/global/6m?f_pango_lineage=BF.10” (accessed on 9 September 2022). (**top**) All major clades; (**bottom**) focus on BF10 lineage.

In the alignments of the RdRp domain and the spike protein, shown in Figure 4 and Supplementary Figure S1, respectively, the numbering is respected and each mutation is easily identified as silent or otherwise. In the RdRp example, the frameshift is discretely managed and one non-synonymous mutation (P323L) is found in the RdRp domain. In the spike example, several non-synonymous mutations were found: T19I, L24-, P25-, P26-, A27S, H69-, V70-, G142D, V213G, G339D, S371F, S373P, S375F, T376A, D405N, R408S, K417N, N440K, L452R, S477N, T478K, E484A, F486V, Q498R, N501Y, Y505H, D614G, H655Y, N679K, P681H, N764K, D796Y, Q954H, N969K. All of this indicates that this spike is deviant from the original strain. A quick request to the PANGOLIN web service confirms the lineage: BF10, corresponding to 22B-like (Omicron variant). Indeed, according to nextstrain, the clade 22B includes the lineage BF10, as presented in Figure 5.

## 4. Discussion

In its final stage, VIMVer is designed as a transversal tool. It should target a wide audience from research studies to clinical applications. It could be implemented as an automatized survey workflow synergized with wet-lab applications and resources, such as the database on RNA viruses described in the VAZyMolO [11] database used in structural and functional studies [12,13,14,15,16,17,18,19]. The application of VIMVer in the research lab is quite straightforward, as it allows the technician, engineer, or researcher to analyze their data straight out of the sequencer. It provides fundamental insight that allows us to sort out the emergence of new data. This quick identification can provide rapid annotation for new genomes which can be exploited by the epidemiologist or medical expert. The double nucleic/proteic interface facilitates the interpretation of a given mutation for any biologist that is neither a biochemist nor bioinformatics expert. Further developments concerning the online VIMVer include extending the reference database to the annotated sequences of VAZyMolO. The latter is a highly curated database of soluble functional or structural protein domains. The combination of both would then allow us to not only spot an emerging mutation but also to correlate it with structural information and compare it within a defined domain family.

The connection of VIMVer to protein structure analysis software, such as AlphaFold [20] or other structural prediction and analysis tools, should facilitate the connection of genotype to phenotype for emerging virus gene products. The main spirit of VIMVer is to propose a deep analysis of each mutation found in the user’s genomic sequence of interest against the corresponding completely annotated reference. Considering its specific conception, VIMVer diverges from existing web services, such as NextClade [3], which does not provide a specific annotated protein-related dataset.

## 5. Conclusions

Viral Instant Mutation Viewer (VIMVer) is a sequence analysis tool of potential use to the community deciphering genomic sequences and connecting them to the phenotype. Our workflow allows the instant visualization of SARS-CoV-2 mutations on both genomic and protein sequences with proper numbering. It delivers an interactive alignment and allows rapid lineage identification that could be easily used in genomic and epidemiological reports. VIMVer can be easily implemented in a larger infrastructure or adapted to all RNA viruses and is interoperable with already existing workflows, as it is able to deliver results in a few seconds, even using a modest computational infrastructure. At the time of writing, the pandemic is now over but still drives us to better prepare ourselves for the next episode. Thus, VIMVer contributes to existing services to better serve the scientific community and allow it to quickly analyze mutational changes detected in the enormous wealth of data generated daily.

## Data Availability

VIMVer is available through a web service (https://vimver.afmb.univ-mrs.fr/). The code and workflow documentation is available at https://github.com/wildevince/VIMVer (accessed on 19 April 2022). The project is under a free software license CeCILL (see more: http://www.cecill.info).

## Supplementary Materials

The following supporting information can be downloaded at: https://www.mdpi.com/article/10.3390/v15081628/s1, Figure S1: Alignment of the GISAID entry EPI_ISL_14769247 on spike protein against Wuhan reference (NC_045512.2).

## Abbreviations

The following abbreviations are used in this manuscript:

VIMVer	Viral Instant Mutation Viewer
SARS-CoV-2	Severe acute respiratory syndrome coronavirus 2
COVID-19	Coronavirus disease 2019
CoV	Coronavirus
NCBI	National center of biotechnology information
GISAID	Global Initiative on Sharing All Influenza Data
NGS	next-generation sequencing
VOCs	variants of concern
ORF#	open reading frame #number
pp#	polyprotein #number
nsp#	non-structural protein #number
ssl	Secure Sockets Layer certificate
RdRp	RNA-dependant RNA-polymerase
RNA	ribonucleic acid
