# Supplementary figures and images for: Viral Instant Mutation Viewer: A Tool to Speed Up the Identification and Analysis of New SARS-CoV-2 Emerging Variants and Beyond
Source: Viruses. 2023 Jul 26;15(8):1628. doi: 10.3390/v15081628 (PMC10458308; doi:10.3390/v15081628)

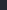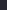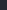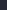

Supplement: Supplementary file 1 [file viruses-15-01628-s001.zip › viruses-2510230-supplementary.pdf]
